# Blind Health Teachers

**Published:** 1872-04

**Authors:** 


### BLIND HEALTH TEACHERS.

The public is indebted to such men as Graham, and Alcott, and Shew, all of them dead now, for many things they wrote in favor of living regular and temperate lives. The Water Cure Journals of the country have disseminated much valuable information, in popular language, in reference to intemperance, the hurtful effects of the use of tobacco, the advantages of personal cleanliness, of ventilation, and of giving judicious attention to the laws of our being, as a means of preserving health, and of regaining it when lost.

Among these publications are "The Phrenological Journal," "The Laws of Life," "The Herald of Health," and "The Health Reformer." Samuel R. Wells, the editor of the "Phrenological Journal," is the father and leader of them all. He has labored long and usefully in the field of temperance, industry, and a wise life, on his pet subject of phrenology. We offer no criticism; its general laws are undoubtedly true; in the application of them, humanity is liable to error. Drs. Trall, and Jackson, and Miller, and Holcombe, and Mrs. Doctor Austin and Lozier, have also disseminated much valuable instruction as to the best means of preserving the human body in perfect function, to a serene old age. But in their wars against the use of medicine, their opposition to vaccination, their admission into their journals of the writings of young, irresponsible, and ignorant persons, from the stand-points of crude opinions, of limited observation, of contracted experience, of puerile logic, and hasty generalization, they have contributed to the perpetuation of much that is plausible but deceptive, and of more that is untrue, and hurtful, and dangerous.

Men of prominence are paid to write on health subjects, more for the value of their names and notoriety, than for the reliability of their teachings. We have in our mind's eye at this moment a brilliant, prolific, but erratic writer, a gentleman of unusual ability, with a clerical education, great opportunities, and much brass, who became a frequent contributor to newspapers and magazines on the subject of health. He soon got on a hobby, a coal of fire, — that is, there had been fire in it once, but it had gone out, and left nothing but an unsightly, soiling lump of black; chemistry calls it "carbon." Our friend made it out that the great regulator of the health and lives of men was the proper supply of "carbon;" but that everybody was taking too much of it, and if people didn't quit, they would catch on fire, and the whole world would be burned to a cinder before anybody could say "Jack Robinson." He went on to explain that the way in which folks took more carbon into their systems than was wholesome, was in eating too much carbonaceous food; then he explained what foods and fluids had most carbon in them. Pigs had a wonderful sight of it; therefore it was wrong to eat pork. Whiskey was all carbon, hence some who drank a good deal of it took fire themselves, and fired away at their wives, their children, and their best friends, with whatever they had in their hands, with the effect that a whole family would be sometimes found sprawling on the floor at once. Then he made a tilt at buckwheat-cakes and molasses; they were almost all carbon, hence buckwheat-cakes and molasses were dreadful, were like tinder in the neighborhood of a flaming volcano. Next, butter and sugar came in for a due share of hygienic anathema. But he forgot to explain at this point why a Greenlander, who could eat twenty pounds of tallow candles at a meal, or swallow two or three gallons of melted lard in a day, and feel the better for it, did not set the North pole on fire, burn up all the freeze in the universe, and thus give everlasting summer to everybody. He went on blazingly until he came down to the hard-pan fact that nobody ought to eat anything but apples and pop-corn. Just at this time he got sick; he became disgusted with the United States of America, and sailed for foreign climes, with bag and baggage.

Not long ago we saw a letter of his in the "New York Independent," in which he had occasion to refer to some of his former scribbling about health and disease, closing with a very suggestive announcement, "but I was an ass then;" and how much "ass-er" must multitudes of his former compeers in the same field be, whose compositions, if printed as written, would show their blissful ignorance of orthography, etymology, syntax, and prosody.

A young eclectic physician once assured us that there had been neither tea, coffee, flesh, fish, nor fowl on his table for five years. Three years later, he had secured for himself a very lucrative practice in Cincinnati; he was going day and night. "How about the coffee, Doctor, and the roast beef?"

"O, I found out that I was mistaken. I couldn't keep up. I drink a cup of very strong coffee at each meal, and it does me good, it makes me strong; and with plenty of exercise, which I have, I find that roast beef and porter-house steak, especially the tender-loin part, makes the best blood in the world."

These things show that the only reliable teachings in reference to health and disease are found in the writings of educated men, whose principles have been founded on the observation of the learned in all ages, from Hippocrates, the father of medicine, born nearly five hundred years before the Saviour's advent, down to the present time, known as the "old school" or "allopathic doctors." The eclectics, the water-cure people, the vegetarians, and the globule men, have never discovered anything; all they really know is learned from the books of allopathic writers. However, they will have "their day," to go out in the night of forgetfulness, leaving nothing of truth in their whole literature, except what was taken from the "old school."

